# Supplementary material for: Relation between lymphocyte to monocyte ratio and survival in patients with hypertrophic cardiomyopathy: a retrospective cohort study
Source: PeerJ. 2022 Mar 29;10:e13212. doi: 10.7717/peerj.13212 (PMC8973459; doi:10.7717/peerj.13212)
Supplement: Supplemental Information 2 [file peerj-10-13212-s002.docx]

Code

1. Sex: 1 = male, 2 = female
2. Hcmfh (Family history of hypertrophic cardiomyopathy): 0 = no, 1 = yes
3. Scdfh (Family history of sudden cardiac death): 0 = no, 1 = yes
4. Chestpain (chest pain): 0 = no, 1 = yes
5. Palpitation: 0 = no, 1 = yes
6. Syncope: 0 = no, 1 = yes
7. Dyspnea: 0 = no, 1 = yes
8. Hypertension: 0 = no, 1 = yes
9. Diabetes: 0 = no, 1 = yes
10. Prete (prior thromboembolism): 0 = no, 1 = yes
11. Vascular disease: 0 = no, 1 = yes
12. Af (atrial fibrillation): 1 = no, 2 = yes
13. Aspirin: 0 = no, 1 = yes
14. Beta (beta blocker): 0 = no, 1 = yes
15. Clopidogrel: 0 = no, 1 = yes
16. Aceiarb (angiotensin converting enzyme inhibitor/angiotensin receptor blocker): 0 = no, 1 = yes
17. Intervention (obstruction intervention): 0 = no, 1 = Alcohol septal ablation, 2 = Septal myectomy
18. Devices: 0 = no, 1= pacemaker, 2 = ICD.
19. Lvoto (left ventricular outflow tract obstruction): 0 = no, 1 = yes
20. Acm (all-cause mortality): 0 = survival, 1 = death
21. Hcmrelateddeath (HCM related death): 0 = survival, 1 = death
22. Hfdeath (heart failure related death): 0 = survival, 1 = death
23. Strokedeath (stroke related death): 0 = survival, 1 = death
24. Scd (sudden cardiac death): 0 = survival, 1 = death.
