# Supplementary material for: Relation between lymphocyte to monocyte ratio and survival in patients with hypertrophic cardiomyopathy: a retrospective cohort study
Source: PeerJ. 2022 Mar 29;10:e13212. doi: 10.7717/peerj.13212 (PMC8973459; doi:10.7717/peerj.13212)
Supplement: Supplemental Information 3 — Abbreviations as in table 1. *: significant p values [file peerj-10-13212-s003.docx]

| Supplementary table 1. Fully adjusted Cox regression analysis of all-cause mortality | | | | | | |
| --- | --- | --- | --- | --- | --- | --- |
| Variable | Change | LMR by tertiles | |  | LMR as continuous variable (< 6.5) | |
|  |  | HR (95% CI) | p value |  | HR (95% CI) | p value |
| Gender | female vs. male | 0.81 (0.39-1.66) | 0.561 |  | 1.26 (0.56-2.83) | 0.576 |
| Family history of SCD | yes vs. no | 2.06 (0.67-6.33) | 0.207 |  | 2.37 (0.75-7.46) | 0.141 |
| Syncope/pre-syncope | yes vs. no | 0.58 (0.28-1.18) | 0.130 |  | 0.59 (0.28-1.25) | 0.169 |
| Dyspnea | yes vs. no | 1.48 (0.70-3.14) | 0.301 |  | 1.07 (0.49-2.34) | 0.871 |
| Atrial fibrillation | yes vs. no | 1.66 (0.80-3.42) | 0.173 |  | 1.49 (0.70-3.21) | 0.302 |
| Resting LVOTG ≥ 30 mm Hg | yes vs. no | 1.69 (0.84-3.43) | 0.144 |  | 1.40 (0.67-2.94) | 0.373 |
| Age (years) | per 1-SD increase | 1.29 (0.91-1.82) | 0.154 |  | 1.45 (0.96-2.17) | 0.077 |
| Hemoglobin (g/L) | per 1-SD increase | 0.76 (0.55-1.05) | 0.096 |  | 0.85 (0.58-1.23) | 0.383 |
| TG (mmol/L) | per 1-SD increase | 0.69 (0.42-1.13) | 0.138 |  | 0.70 (0.44-1.12) | 0.136 |
| LDL-C (mmol/L) | per 1-SD increase | 0.66 (0.47-0.94) | 0.020* |  | 0.64 (0.44-0.94) | 0.022* |
| LA diameter (mm) | per 1-SD increase | 1.22 (0.87-1.71) | 0.244 |  | 1.36 (0.94-1.97) | 0.102 |
| MWT (mm) | per 1-SD increase | 1.21 (0.85-1.71) | 0.294 |  | 1.46 (1.00-2.15) | 0.052 |
| LVEF (%) | per 1-SD increase | 0.77 (0.55-1.06) | 0.107 |  | 0.71 (0.51-0.98) | 0.037^*^ |
| LMR | per 1-SD increase | - |  |  | 0.38 (0.21-0.68) | 0.001^*^ |
| LMR |  |  |  |  |  |  |
| tertile 1 |  | Ref. |  |  |  |  |
| tertile 2 |  | 0.43 (0.20-0.91) | 0.027^*^ |  |  |  |
| tertile 3 |  | 0.39 (0.17-0.90) | 0.028^*^ |  |  |  |

Abbreviations as in table 1.

*: significant p values
